# Electrophysiological comparison of left versus right stellate ganglia neurons

**DOI:** 10.1016/j.jmccpl.2025.100293

**Published:** 2025-03-20

**Authors:** Arie O. Verkerk, Carol Ann Remme, Molly O'Reilly

**Affiliations:** a Department of Experimental Cardiology, Amsterdam University Medical Center, University of Amsterdam, Meibergdreef 9, 1105, AZ, Amsterdam, the Netherlands; b Department of Medical Biology, Amsterdam University Medical Center University of Amsterdam, Meibergdreef 9, 1105, AZ, Amsterdam, the Netherlands

## Abstract

**Background:**

The stellate ganglia of the peripheral autonomic nervous system innervate the heart and continuously fine-tune cardiac function to meet physiological demands. The right stellate ganglion (RSG) predominantly innervates the sinoatrial node and has functional effects on chronotropy/heart rate, whereas the left stellate ganglion (LSG) has predominance in the ventricular myocardium and impacts inotropy/contractility. Whilst the innervation patterns and functional consequences of block and stimulation are well-documented, basic electrophysiological characterisation and single-cell comparison of RSG and LSG neurons has not been performed. In addition, sex differences in stellate ganglion action potential (AP) parameters may exist, but remain as yet unknown.

**Methods/results:**

Here we characterise the electrical properties of enzymatically isolated mouse stellate ganglia neurons using the patch clamp technique. Using 500 ms depolarising pulses of varying amplitude, we provide detailed characterisation of basic AP properties and their correlations. We reveal that there are two populations of neurons in terms of their AP firing properties (phasic or tonic firing), with the majority (65 %) firing with a phasic pattern. When all recordings were pooled, tonic neurons had a significantly larger AP amplitude (85 ± 3.0 vs 76 ± 2.4 mV) and overshoot (28 ± 1.8 vs 19 ± 1.8 mV) compared to phasic neurons (*P* < 0.05). Moreover, phasic neurons did not fire spontaneously, whereas 50 % of tonic neurons did, and more often presented with anodal break excitation (*P* < 0.05). When male vs female neurons were compared (with LSG and RSG neurons as subgroups), males had a more negative minimum diastolic potential (MDP; −55 ± 1.7 vs −47 ± 3.0 mV, *P* < 0.05) and higher percentage of anodal break excitation (*P* ≤ 0.05). When LSG vs RSG neurons were compared (with gender as subgroups), no significant differences were observed except a higher percentage of anodal break excitation in the RSG (*P* ≤ 0.05).

**Conclusions:**

Isolated RSG and LSG neurons have similar AP firing patterns and properties. A significant difference was observed in the MDP and anodal break excitation of male vs female neurons. However, all other AP parameters were similar. This suggests that the LSG and RSG can be combined irrespective of sex when investigating the electrophysiological properties of these distinct anatomical structures.

## Highlights

• A first-of-its-kind comparison of isolated left vs right and male vs female stellate ganglion neuron (SGN) electrical properties.
• Identification of gender-specific differences in the minimum diastolic potential and anodal break excitation of SGNs without major differences between left and right SGNs.
• A simplified protocol for SGN isolation and culture.

## Graphical abstract

**Figure:**
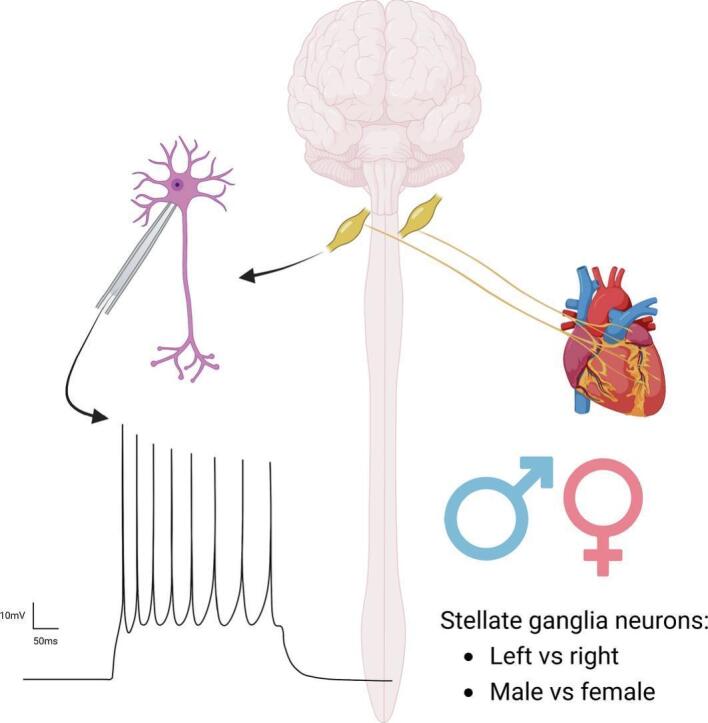

## 1 Introduction

The stellate ganglia are vitally important anatomical structures in respect to cardiac function. They are part of the peripheral autonomic nervous system, located alongside the spinal cord, and contain the vast majority of the sympathetic neurons that project directly to the heart. During any instances of sympathetic activity (exercise, emotion, or fight-or-flight response) the neurons of the stellate ganglia, and their cardiac-projecting neurites, become activated. This fine-tunes the functioning of the heart, enhancing sympathetic tone, modifying neurotransmitter release, and increasing cardiac output to meet the demands of the new physiological state.

In numerous cardiac diseases, the stellate ganglia are recognised as a pathophysiological contributor and thus a target for treatment. In inherited arrhythmia syndromes such as Catecholaminergic Polymorphic Ventricular Tachycardia (CPVT) and Long-QT Syndrome (LQTS), surgical removal of the stellate ganglia (stellectomy) is a known treatment approach that yields positive outcomes [[1], [2], [3]]. Moreover, recent fundamental studies reveal there to be functional alterations of the stellate ganglia in prohypertensive [4], prehypertensive [5], and CPVT [6] animal models.

The right and left stellate ganglia have overlapping but also distinct cardiac innervation patterns. The right stellate ganglion (RSG) has predominant innervation of the pace-setting sinoatrial node of the heart, whereas the left stellate ganglion (LSG) predominantly innervates the ventricular myocardium. Consequently, the ganglia have differing functional effects in terms of their primary physiological impact on chronotropy/heart rate (RSG) or inotropy/contractility (LSG) [[7], [8], [9]].

Despite recognition of their differing physiological roles, few studies have investigated the LSG and RSG in detail, and often in studies of disease models neurons from the LSG and RSG are pooled. Whilst the innervation patterns and functional consequences of block and stimulation are well-documented [[10], [11], [12]], as well as their neurochemical profiles [13], basic electrophysiological characterisation and comparison of single cell RSG and LSG neurons is currently lacking. Moreover, a transcriptomic study of the LSG has demonstrated sex differences in the expression of genes that encode ion channels [13]. However, any resulting electrical differences have as yet not been investigated.

This study aims to fill this knowledge gap by providing action potential (AP) characterisation and comparison of the LSG and RSG in both male and female mice - investigating whether electrical differences exist and if it is appropriate to pool the two distinct anatomical structures when performing comparative disease studies.

## 2 Methods

### 2.1 Animals

Wild-type C57Bl6j mice were used for the experiments detailed herein - male and female, 2–6 months. Housing, handling, and experiments were performed in agreement with the Institutional guidelines (animal license number 18–4986).

### 2.2 Stellate ganglion neuron isolation

Following terminal anaesthesia (4 % isoflurane inhalation in O_2_), the RSG and LSG were localised, separately removed and placed in ice-cold PBS. Single RSG and LSG neurons were isolated by an enzymatic dissociation procedure. Ganglia were transferred to a nominally Ca^2+^-free Tyrode's solution (20 °C) (pH 7.4; NaOH) containing (in mM): NaCl 140, KCl 5.4, CaCl_2_ 0.01, MgCl_2_ 1, glucose 5.5, HEPES 5 as well as Liberase TM (26 U/ml) and Elastase (211 U/ml) enzymes. The tissue was gently agitated in a shaking water bath at 37 °C for 28 min. Subsequently, ganglia were washed in nominally Ca^2+^-free Tyrode's solution (20 °C), and thereafter in normal Tyrode's solution (20 °C) (pH 7.4; NaOH) containing (in mM): NaCl 140, KCl 5.4, CaCl_2_ 1.8, MgCl_2_ 1, glucose 5.5, HEPES 5. Finally, ganglia were transferred to B-27 Plus Neuronal Culture System (Gibco) media (20 °C) and single cells were obtained by manual trituration using fire-polished glass pipettes. Single cells were plated on coverslips coated with 100 μg/ml poly-d-lysine and 10 μg/ml laminin. Neurons were left to adhere in a 37 °C incubator overnight before experiments were performed the subsequent day. Previous work has shown that such a short culturing period does not affect the electrical phenotype of stellate ganglia neurons [5]. 2 mice were used for each isolation. Measurements were only included in analysis when recordings were obtained from both the left and right stellate from the same isolation. A total of 6 different isolations were included in the analysis.

### 2.3 Patch clamp electrophysiology

Coverslips were transferred to a recording chamber and were continually superfused with normal Tyrode's solution (37 °C). APs were recorded using the amphotericin perforated patch clamp technique with an Axopatch 200B amplifier (Molecular Devices, Sunnyvale, CA, USA). Data acquisition was realized using a CED micro1401 driven by custom-made acquisition software (Axograph) and data analysis was performed with custom software (MacDaq, version 15.4; kindly provided by Antoni C. G. van Ginneken). Signals were low pass filtered with a cut-off frequency of 5 kHz and digitized at 50 kHz. Patch pipettes were pulled from borosilicate glass (TW100F-3, World Precision Instruments Germany Gmb) using a vertical microelectrode puller (PC-100; Narishige Scientific Instrument, Japan) and had tip resistances of 1.5–3 MΩ after filling with the pipette solution as indicated below. All potentials were corrected for the estimated liquid junction potential [14].

**Fig. 1:**
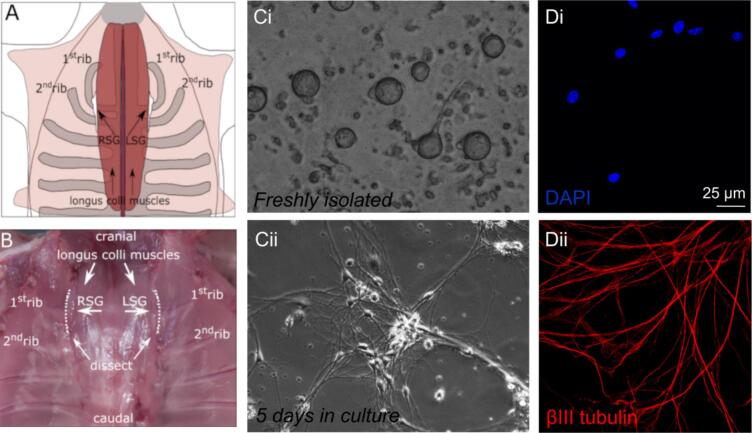
**Isolation, culturing and immunostaining of stellate ganglion neurons.** A and B, Schematic drawing (A) and microscopic view (B) of the location of the stellate ganglia – adapted from [16]. C, Light microscopy showing the appearance of freshly isolated stellate ganglia neurons (Ci) and those neurons cultured for 5 days (Cii). D, Confocal microscopy of cultured stellate ganglia neurons stained for DAPI (Di, blue) and βIII tubulin (Dii, red). Scale bar is 25 μm. (For interpretation of the references to colour in this figure legend, the reader is referred to the web version of this article.)

Patch pipettes were filled with a solution containing (in mM): K-gluconate 125, KCl 20, NaCl 5, amphotericin-B 0.44, HEPES 10, pH 7.3 (KOH). APs were evoked by 500 ms depolarising current pulses of varying amplitude (0–200 pA, in 50 pA steps). We counted the number of APs during a 500 ms stimulus. Further, we analysed AP parameters as described previously [15] and cells which did not overshoot the zero potential level were excluded. The resting membrane potential (V_rest_) was defined as the potential immediately before the depolarising pulse. From the first AP of the 150 pA depolarising pulse, we measured the: AP overshoot, AP amplitude (APA) as the difference between V_rest_ and overshoot, maximal AP upstroke velocity (V_max,dep_), maximal AP repolarisation velocity (V_max, rep_), AP duration (APD) at 50 % repolarisation (APD_50_) and the minimum diastolic potential (MDP) as the most negative hyperpolarisation voltage following the first AP. These AP parameters as well as the cycle length were in a subset of cells also characterized from 20 subsequent APs during 150 ms depolarising pulses. Finally, we tested anodal break excitation, i.e. AP generation after a hyperpolarising pulse, by a 100 pA, 500 ms hyperpolarising pulse. All analysed AP parameters are schematically indicated in Fig. 1A.

### 2.4 Statistics

Data are expressed as dot plots, percentage, or as average ± SEM. Each single cell recording using patch clamp methodology is considered as a biological replicate, since the data are from: a) single cells, b) one isolation is performed with 2 mice, c) and the variation of single cell recordings within isolations is typical larger than between isolations. Statistical analysis was carried out with SigmaStat 3.5 (Systat Inc., St. Louis, MO) and GraphPad Prism (version 10.2.0 (392)). Normality and equal variance assumptions were tested with the Kolmogorov-Smirnov and Levene median tests, respectively. Two groups were compared using the Fisher exact test, unpaired *t*-test, or nested t-test. Multiple logistic regression is used for nested categorized data which was transformed into binary variables. One-way and Two-way repeated measures (RM) ANOVA, followed by the Students-Newman-Keuls post hoc test were used to test the significance for the data in Fig. 4D. Friedman RM ANOVA on ranks followed by the Students-Newman-Keuls post hoc test was used for Fig. 5. Linear relationships were analysed using the Pearson correlation coefficient (R) and significance level. Weak, moderate and strong relationships were defined R values of <0.3, 0.3–0.5, and 0.5>, respectively. *P* < 0.05 was considered statistically significant.

## 3 Results

### 3.1 Stellate ganglion neuron preparation

Left (LSG) and right (RSG) stellate ganglia were localised and removed separately from each mouse (Fig. 1A and B), as previously described [16]. Single neurons were obtained by an enzyme digestion method (Fig. 1Ci) and were subsequently cultured (Fig. 1Cii, Di, and Dii). The isolated cells regrew characteristic neuronal structures (axons and dendrites) when in culture, and were positive for DAPI and βIII tubulin - indicating that the isolated cells were indeed stellate ganglia neurons.

### 3.2 Basic action potential properties of stellate ganglion neurons

#### 3.2.1 Overview of AP parameters

**Fig. 2:**
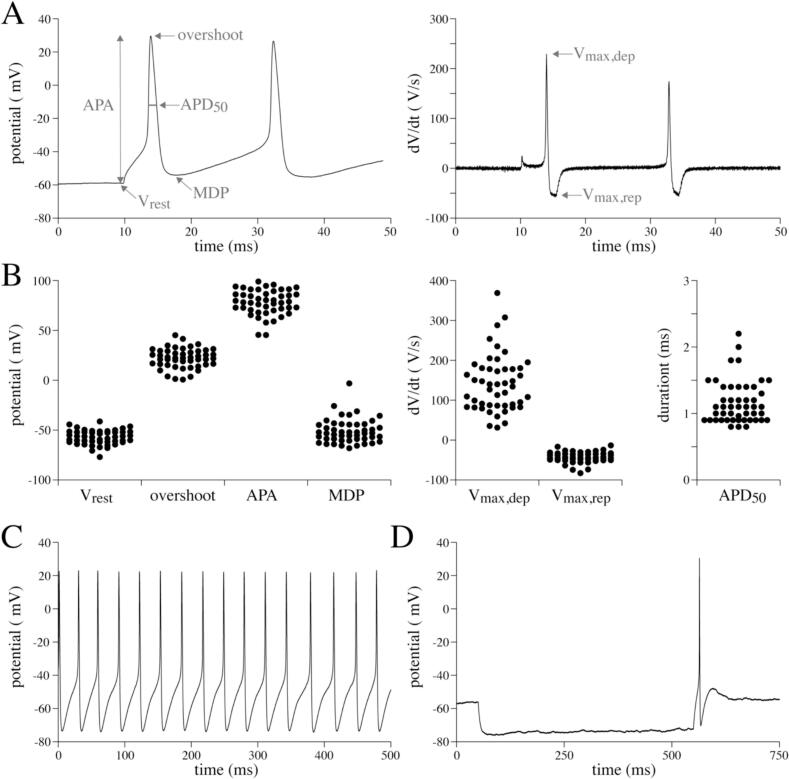
**Basic action potential (AP) characteristics of stellate ganglion neurons.** A, Example of APs evoked by a 150 pA depolarising pulse (left panel) and the first derivative of these APs (right panel). The analysed parameters are schematically indicated. B, Dot plots of all AP parameters of 48 single neurons from 12 cell isolations. Each single cell recording is considered as an independent measure (see Methods section). C, Typical example of spontaneous APs (without current injection). D, Typical example of anodal break excitation in response to a 100 pA hyperpolarising pulse. V_rest_ = potential 5 ms before the first AP, APA = AP amplitude, MDP = maximal diastolic potential after the first AP, V_max,dep_ = maximal AP upstroke velocity, V_max,rep_ = maximal velocity of AP repolarisation, APD_50_ = AP duration at 50 % of repolarisation.

In Fig. 2, we summarized the general AP properties of all measured stellate ganglion neurons. For this purpose, all 48 recordings obtained from LSG and RSG neurons of both male and female mice were pooled. In later sections, we focussed in more detail on potential gender and left vs right differences. Fig. 2A, left panel, shows typical AP recordings evoked by a 150 pA, 500 ms long depolarising pulse, i.e., the pulse where all measured cells evoked at least one AP. Fig. 2A, right panel, shows the first derivative of the AP, reflecting the change in voltage per second (dV/dt). Analysed AP parameters are schematically indicated. Fig. 2B depicts dot plots of the analysed AP properties of the first evoked AP. V_rest_ was between −40 and − 80 mV and overshoot ranged between 0 and 50 mV. Consequently, APA was typically 50 to 100 mV. V_max,dep_ was quite variable with values between 30 and 400 V/s, indicating that the main inward current underlying the AP upstroke, i.e., the sodium current, may differ largely between neurons. V_max,rep_, due to outwardly directed potassium currents, was between −30 and − 70 V/s. The APD_50_ was typically short and ranged between 0.8 and 2.5 ms. MDP was slightly more depolarised than the V_rest_. Without current injection, we found that 7 out of 48 neurons (14.6 %) showed spontaneous AP generation, with typical examples shown in Fig. 2C. Moreover, anodal break excitation in response to a 100 pA hyperpolarisation pulse was observed in 20 out of 48 neurons (41.7 %; Fig. 2D).

#### 3.2.2 Relationships between AP parameters

**Fig. 3:**
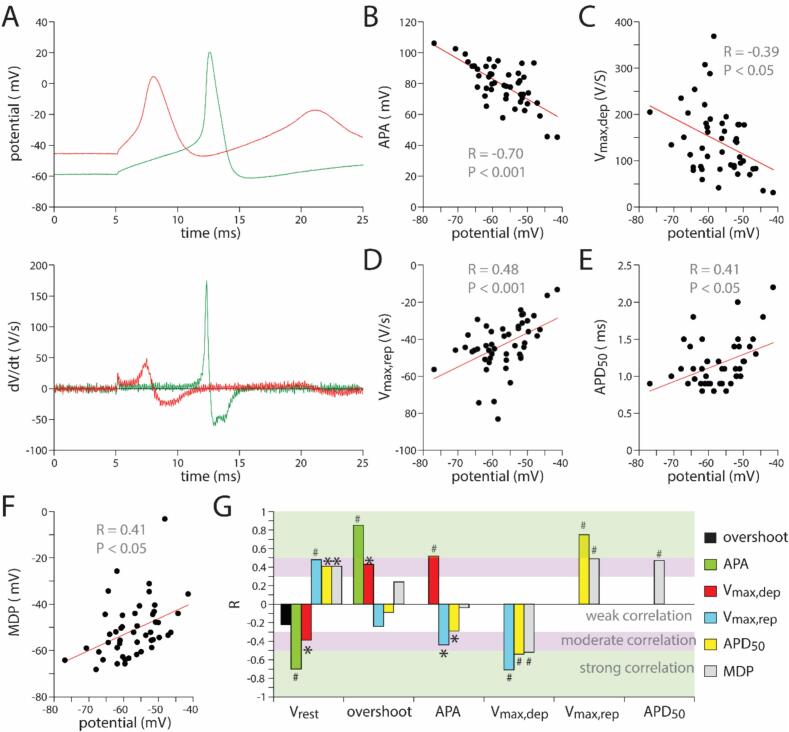
**Correlations between AP parameters.** A, Two examples of APs with different V_rest_ evoked by a 150 pA depolarising pulse (top panel) and the first derivative of these APs (bottom panel). B to F, Linear relationships (red solid lines) of V_rest_ with APA (B), V_max,dep_ (C), V_max,rep_ (D), APD_50_ (E), and MDP (F) of 48 neurons. Each single cell recording is considered as an independent measure (see Methods section). R indicates the Pearson correlation coefficient. G, R values of all linear relationships between AP parameters. Green, pink and white horizontal bars indicate the strength of the correlations. *P < 0.05, ^#^*P* < 0.001. (For interpretation of the references to colour in this figure legend, the reader is referred to the web version of this article.)

It is evident from Fig. 2B that there is some variation in AP parameters between neurons. Fig. 3A shows typical AP recordings from two separate neurons obtained from the same RSG neuron isolation, displaying a clear difference in V_rest_ between both cells. The AP with the most negative V_rest_ had the fastest AP upstroke velocity, but also the fastest repolarisation and shortest AP. In addition, the MDP in this cell was more negative compared to the other depicted neuron. Next, we determined if this is a consistent finding by testing for relationships between AP parameters. Therefore, we plotted linear fits and performed analysis of the Pearson correlation coefficients (R) and significance (Fig. 3B-G). All R's between the AP properties are summarized in Fig. 3G, and it is evident that there are multiple strong and moderated linear associations between the variables. For example, the V_rest_ shows a linear relationship with the AP amplitude (APA) with an R of −0.70 (*P* < 0.001) (Fig. 3B,G), thus indicating a strong negative correlation. This may be partially related to our definition of APA, since we defined it as the difference between V_rest_ and overshoot. More importantly, V_rest_ also shows linear correlations (*P* < 0.05) with V_max,dep_ (Fig. 3C,G), V_max,rep_ (Fig. 3D,G), APD_50_ (Fig. 3E,G) and MDP (Fig. 3F,G), although these can be considered as moderate. These correlations are likely caused by neuronal membrane current properties. V_rest_ affects importantly the sodium current responsible for the AP upstroke, and a more depolarised V_rest_ will result in less available sodium channels since many channels will be in the inactivated state. As a result, V_max,dep_ will be smaller. Similarly, a more depolarised V_rest_ will reduce the availability of the I_A_ current, i.e., the transient outward potassium current, which is an important repolarising current in neurons. Consequently, the V_max,rep_ becomes smaller resulting in longer APs with more positive MDPs, as indicated by the strong correlations of these parameters.

There is also a strong negative correlation between V_max,dep_ and V_max,rep_ (Fig. 3G), but as mentioned above this is likely due to their underlying channel properties and the common relation with V_rest_.

#### 3.2.3 AP firing patterns

**Fig. 4:**
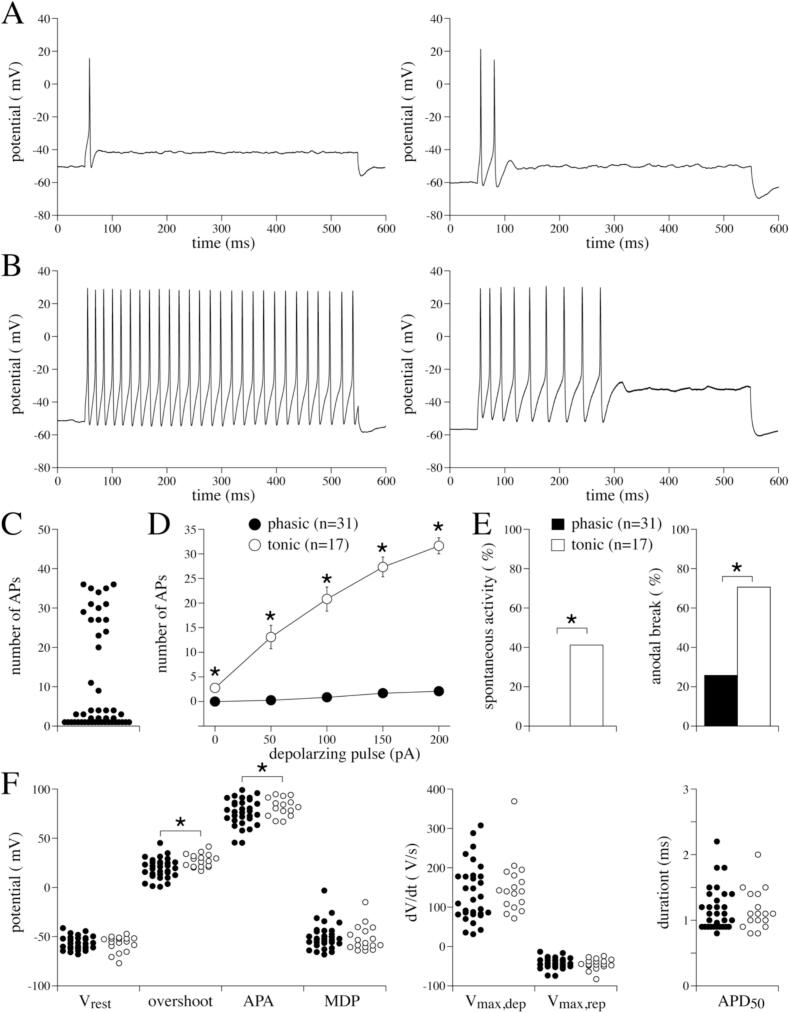
**AP parameters of phasic and tonic neurons.** A, Two examples of phasic firing evoked by a 150 pA, 500 ms depolarising pulse. B, Two examples of tonic firing evoked by a 150 pA, 500 ms depolarising pulse. C, Dot plot of the number of APs during the 150 pA, 500 ms depolarising pulse of 48 neurons, Each single cell recording is considered as an independent measure (see Methods section). D, Number of APs vs the depolarising pulses for phasic and tonic neurons. Data are average ± SEM. *P < 0.05 (Two-way RM ANOVA). E, Percentages of phasic and tonic neurons with spontaneous APs and anodal break excitation *P < 0.05 (Fisher exact test). F, Dot plots of the AP parameters in phasic (*n* = 31; closed circles) and tonic (*n* = 17, open circles) neurons. *P < 0.05 (unpaired *t*-test). ‘n’ indicates the number of cells; phasic firing was found in 6 out of 6 isolations, tonic firing in 5 out of 6 isolations.

In the sections above, we focused on the first AP evoked by the 150 pA depolarising pulse. However, looking into the firing pattern during the complete 150 pA depolarising pulse in more detail revealed two distinct populations of neurons, as shown in the typical AP examples of Fig. 4, A and B. The majority of the neurons (31 out of 48) fired with a ‘phasic’ pattern – firing one or a small number of APs (<4, Fig. 4A and C) before becoming quiescent for the rest of the 150 pA, 500 ms depolarising pulse. The remaining 35 % of the neurons fired with a ‘tonic’ pattern – firing almost continuously throughout the same depolarising stimulus (Fig. 4B, left panel) and the number of APs during the 150 pA, 500 ms depolarising pulse is typically >9 (Fig. 4C). If the number of APs in a tonic firing cell is at the lower end, the firing ceased in the course of the depolarizing pulse, as shown in Fig. 4B, right panel. The firing frequencies of both the phasic and tonic neurons significantly increased in response to increasing depolarising pulses (*P* < 0.05, One-way RM ANOVA), but that of phasic neurons remained relatively low at all depolarising pulses (Fig. 4D). Consequently, phasic and tonic firing patterns differ significantly (*P* < 0.05, Two-way RM ANOVA) at all tested depolarising pulses (Fig. 4D). Interestingly, phasic neurons did not fire spontaneously, whereas 42 % of tonic neurons did fire spontaneously without any current injection (7 out of 17 cells) (Fig. 4E). The number of cells with anodal break excitations was also significantly higher in tonic neurons (Fig. 4E) (*P* < 0.05). Phasic and tonic firing neurons were observed in all isolations, except in one where only phasic neurons were found. If present, the amount of tonic firing neurons was equal to half of the amount of phasic firing neurons.

When analysing the parameters of the first AP at the 150 pA depolarising pulses, tonic neurons were found to have a significantly larger overshoot and APA compared to phasic neurons (Fig. 4F) (P < 0.05). All other AP parameters (V_rest_, MDP, V_max,dep_, V_max,rep_ and APD_50_) did not differ significantly between phasic and tonic neurons.

**Fig. 5:**
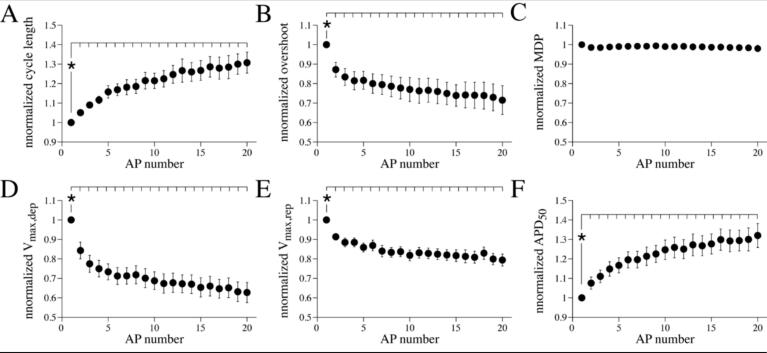
**AP parameters in consecutive APs during a 150 pA depolarising step.** A-F, Changes of cycle length (A), overshoot (B), MDP (C), V_max,dep_ (D) V_max,rep_ (E) and APD_50_ (F) during the first 20 APs of continuously firing tonic cells (*n* = 15). Each single cell recording is considered as an independent measure (see Methods section). Data are average ± SEM and is normalized to the AP of the 1st AP. *P < 0.05 1st vs consecutive APs (Friedman RM ANOVA on ranks).

While we have analysed and compared the first AP of the 150 pA depolarising pulse in Fig. 4F, it is evident from the typical APs shown in Fig. 2A and Fig. 4A and B, that APs may change during the course of the depolarizing pulse, which is a well-known feature of neurons. Next, we characterized these AP changes in detail for isolated stellate ganglia neurons. Therefore, we analysed the AP properties of the first 20 APs of continuously firing tonic cells and plotted the AP properties normalized to the value of the first AP (Fig. 5, A-F)). Cycle length (Fig. 5A) and the APD_50_ (Fig. 5F) progressively increased and reached a steady-state condition after approximately 15 APs. The overshoot (Fig. 5B), V_max,dep_ (Fig. 5D) and V_max,rep_ (Fig. 5E) decreased relatively fast within the first 5 APs, with the most substantial effects between the 1st and 2nd AP. Thereafter, a steady-state condition was reached. The MDP was not affected in course of the depolarizing pulse. The observed changes in the consecutive APs reflect importantly the correlations between the various AP properties (Fig. 3) and are likely due to a more inactivated state of sodium and potassium channels.

#### 3.2.4 AP properties of male versus female stellate ganglion neurons

**Fig. 6:**
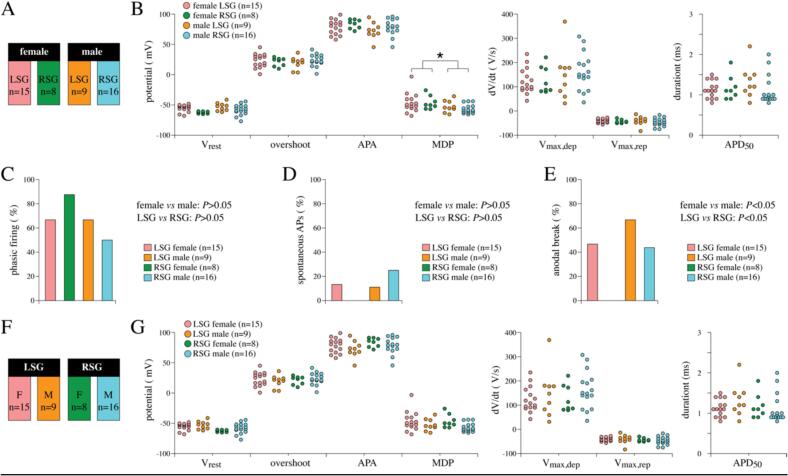
**AP parameters of LSG and RSG neurons of female and male mice.** A, Group design with female and male as main groups and LRS and RSG as subgroups. ‘n’ indicates the number of cells measured and each single cell recording is considered as an independent measure (see Methods section). The colours for the subgroups are used consistently in all panels. B, Dot plots of the AP parameters in female and male neurons (with LRS and RSG as subgroups). MDP in male neurons is significantly different from female neurons without subgroups differences. *P < 0.05 (nested t-test). C-E, Percentage of phasic firing neurons (C), spontaneous APs (D), and anodal breaks (E) of female and male LSG and RSG neurons. *P < 0.05 (multiple logistic regression). F, Group design with LSG and RSG as main groups and gender as subgroups. G, Dot plots of the AP parameters in LSG and RSG neurons (gender as subgroups).

In the above section, we provide a general description of AP properties and firing phenotypes of stellate ganglion neurons and have therefore pooled the AP data of female and male mice as well as LSG and RSG isolations. Next, we tested for sex differences in stellate ganglion neurons. Hence, we compared the basic firing patterns and AP properties of male and female mice, with LSG and RSG isolations as subgroups (Fig. 6A), using nested statistical analyses. The continuous data of AP properties was compared using nested t-test analysis and demonstrated that neurons of male mice have a slightly more negative MDP (*P* < 0.05), without significant differences in the LSG and RSG subgroups (Fig. 6B). All other AP parameters are not affected by sex (Fig. 6B). The categorical variables of phasic vs tonic firing (Fig. 6C), spontaneous vs non-spontaneous (Fig. 6D) and anodal vs no anodal break excitation (Fig. 6E), from male/female and LSG/RSG neurons, were transformed to binary variables for statistical analysis and compared using multiple logistic regression. As shown in Fig. 6, C and D, neither the ratio of phasic neurons (Fig. 6C) nor the number of cells with spontaneous activity (Fig. 6D) was significantly affected by sex. The amount of anodal break excitations was significantly different (*P* ≤ 0.05) in female compared to male, with a lower number of anodal breaks in female, due to the absence of anodal breaks in female RSG neurons (Fig. 6E).

#### 3.2.5 AP properties of left versus right stellate ganglion neurons

Finally, we compared the firing pattern and AP properties of isolated neurons from the LSG and RSG, with gender as subgroups (Fig. 6F). The AP properties of LSG and RSG neurons were not significantly different (Fig. 6G; nested t-test). Analysis using multiple logistical regression demonstrated that anodal break excitation was significantly lower in RSG due to the absence of anodal breaks in female RSG (*P* ≤ 0.05; Fig. 6E), while the ratio of phasic neurons (Fig. 6C) as well as the number of cells with spontaneous AP generation (Fig. 6D) was similar, as already mentioned in the section of gender differences section.

## 4 Discussion

Here we document for the first time the comparative basic electrophysiological profiles of isolated neurons from the left (LSG) versus the right stellate ganglion (RSG) of mice. Detailed analysis of basic action potential (AP) parameters (V_rest_, V_max,dep_, V_max,rep_, APA, overshoot, MDP, and APD_50_) revealed a number of relationships and correlations, as expected from the underlying ionic basis of these measures. We revealed two distinct neuronal populations based on AP firing patterns, namely tonic or phasic type neurons, as has been reported previously in the stellate ganglia of rats [5], and in the sympathetic chain of guinea pigs [17]. We also reveal novel differences in these neuronal subtypes including a heightened propensity for spontaneous AP firing and anodal break excitation in tonic neurons compared to phasic, and significant differences in AP amplitude and overshoot properties. It is known that these neuronal subtypes have different target tissue innervation patterns, in that tonic neurons innervate fewer postsynaptic targets than phasic [18]; these novel electrophysiological characterisations may be important for these innervation differences.

When comparing the basic AP properties of male versus female stellate ganglia neurons, we observed a small difference in the MDP, but not in any other AP parameter. The number of phasic and spontaneous firing neurons was similar, but the number of anodal breaks was lower in female. This corresponds well with recent work showing that estradiol increases two potassium (K^+^) currents (fast inactivating voltage-gated A-type K^+^ channel currents and non-inactivating M-type K+ channel currents) but does not affect spiking excitability [19]. This suggests that the electrical profile of these neurons is hardly affected by sex, and thus the two sexes can be combined when performing comparative studies [6]. However, there is the possibility that there are sex-dependent or sidedness-dependent remodelling in different disease states, so caution should be taken when combining these in disease conditions.

Interestingly, researchers performed RNAseq analysis of the LSG and identified gene expression differences between male and female C57Bl6j mice, including differences in the expression of genes that are important for encoding ion channels, such as *Kcna2* (K_v_1.2) [13]. However, here we show that these ion channel gene expression differences do not translate to differences in the baseline resting membrane potential or the excitability of these cardiac sympathetic neurons, at least under the conditions tested in the present manuscript, as the researchers had speculated.

Lastly, we also compared the basic AP parameters of the LSG and RSG. We found that RSG neurons have similar AP properties and firing patterns (except anodal breaks) as LSG neurons. Thus, electrical properties do not contribute to the different functional roles of the two structures, with the RSG predominantly innervating and affecting the sinoatrial node and heart rate, and the LSG having predominance of ventricular myocardium innervation and contractility. In addition, it demonstrates that LSG and RSG neurons can be pooled in further research.

## 5 Conclusion

Here we show that the electrical profile of neurons of the LSG and RSG of males and females is similar, and thus they can be pooled in future studies. We provide a protocol for the isolation and investigation of mouse stellate ganglia neurons, which will be useful for studies of disease conditions involving genetic factors and evidence of stellate involvement. For example, in inherited arrhythmia conditions such as Catecholaminergic Polymorphic Ventricular Tachycardia (CPVT) and Long-QT Syndrome (LQTS), where stellectomy is a viable treatment option.

### 5.1 Limitations

The underlying ionic basis of the observed AP differences could not be investigated, as one cannot first identify whether a neuron is phasic or tonic (current-clamp configuration) before recording very specific ionic currents (voltage-clamp configuration) with modified pipette and bath solutions. Ionic currents could be measured during net current measurements as drug-sensitive, but that was outside the scope of the current study since AP property and firing patterns differences were minimal. Selective fluorescence labelling of the different types of neurons, if possible, could aid future studies to investigate this further.

## CRediT authorship contribution statement

**Arie O. Verkerk:** Writing – review & editing, Writing – original draft, Visualization, Formal analysis, Conceptualization. **Carol Ann Remme:** Writing – review & editing, Writing – original draft, Supervision, Resources, Funding acquisition, Conceptualization. **Molly O'Reilly:** Writing – review & editing, Writing – original draft, Visualization, Resources, Project administration, Methodology, Investigation, Funding acquisition, Formal analysis, Data curation, Conceptualization.

## Funding

This research was funded by the Dutch Heart Foundation (03-006-2022-0036) and 10.13039/501100001826ZonMw (Off Road grant 04510012110049).

## Declaration of competing interest

Molly O'Reilly reports financial support was provided by Netherlands Heart Foundation. Molly O'Reilly reports financial support was provided by 10.13039/501100001826Netherlands Organisation for Health Research and Development. If there are other authors, they declare that they have no known competing financial interests or persona relationships that could have appeared to influence the work reported in this paper.
